# Brain Injury and Neurodegeneration: Molecular, Functional, and Translational Approach 3.0

**DOI:** 10.3390/biomedicines13123096

**Published:** 2025-12-16

**Authors:** Pankaj Gaur, Meenakshi Ahluwalia, Pankaj Ahluwalia, Kumar Vaibhav

**Affiliations:** 1 Lombardi Comprehensive Cancer Center, Georgetown University Medical Center, Washington, DC 20057, USA; pg684@georgetown.edu; 2 Department of Pathology, Medical College of Georgia, Augusta University, Augusta, GA 30912, USA; meenuahluwalia@gmail.com (M.A.); pahluwalia@augusta.edu (P.A.); 3 Georgia Cancer Center, Medical College of Georgia, Augusta University, Augusta, GA 30912, USA; 4 Brain Injury, Senescence and Translational Neuroscience Lab, Department of Neurosurgery, Medical College of Georgia, Augusta University, Augusta, GA 30912, USA

A brain injury or disease is no longer limited to neuroinflammation or neuronal loss; it has emerged as a multifaceted complex pathology that includes a mechanistic thread of complex events from ischemic/hypoxic injury, glial activation, and metabolic and ionic dysregulation to extracellular vesicle (EV)-mediated signaling, systemic metabolic diseases, psychiatric illness, and neurodegeneration. Hypoxia is one of the critical outcomes of many brain diseases, such as stroke and TBI [1,2,3,4]. In addition, hypoxia is key to the pathogenesis of countless neurological disorders, including Alzheimer’s, Parkinson’s, and other age-related neurodegenerative diseases [5]. Therefore, hypoxia and related molecules can be targeted for therapeutic benefits in brain injury and diseases. In this line, an experimental rat study by Aliyeva et al. (2025) examined the modulation of heat shock protein 70 (HSP70) to counteract neurobehavioral deficits induced by prenatal hypoxia (PH) [6]. Pregnant rats were introduced to sodium nitrite-induced hypoxia, and offspring were treated with various agents, including Angiolin, Cerebrocurin, Tamoxifen, Thiotriazoline, and others. PH caused significant reductions in motor activity, exploration, and memory performance at 1–2 months of age in offspring. Angiolin and Cerebrocurin were most effective at restoring exploratory behavior, emotional stability, learning, and memory [6]. In addition to the oxygen saturation indicator, blood and biofluid biomarkers such as serum growth factor, ions, peptides, cytokines, chemokines, or other damage-associated molecular patterns provide vital clues for diagnosis, prognosis, and recovery in patients. Ionic and electrolyte homeostasis provides an additional nexus between injury and inflammation. An in vitro study by Mahajan et al. (2024) investigated the role of elevated chloride (hyperchloremia) and sodium (hypernatremia) levels on ischemic brain cells [7]. Human microglial (HMC-3) and neuronal (SH-SY5Y) cells were exposed to sodium chloride and sodium acetate under oxygen–glucose deprivation/reperfusion (OGD/R). Both ionic disparities significantly reduced cell viability, minimized NO production, increased cytotoxicity, and altered caspase-1 and 3 expressions. Neuronal survival was especially reduced when exposed to microglial-conditioned media from hyperchloremic or hypernatremic environments [7]. The study suggests that excessive sodium and chloride ions elevate the neuronal injury and lead to poor outcomes in stroke patients with electrolyte disturbances. Clinically, stroke and traumatic brain injury patients with electrolyte imbalances have shown worse outcomes [8,9,10]. Thus, homeostatic ionic regulation represents an underappreciated modulator of neuroinflammation.

Moreover, brain health is also affected by systemic organ dysfunction, and molecular and inflammatory mediators are highly implicated in it. For example, the retrospective study of 92 hemodialysis patients found that elevated serum growth differentiation factor-15 (GDF-15) was strongly associated with cognitive impairment. Patients with cognitive dysfunction (<24 points) had significantly higher GDF-15 levels (7500 pg/mL) than cognitively normal patients (4808 pg/mL, *p* = 0.001). Further, uremic toxin indoxyl sulfate increased GDF-15 expression and neuronal injury in vitro and in vivo and provides a mechanistic link between peripheral organ dysfunction and brain health [11]. With chronic kidney disease (CKD) known to contribute to white matter lesions, brain atrophy, and cognitive decline [12,13,14,15], this evidence reinforces that peripheral disease amplifies brain vulnerability via immune, metabolic, and vascular pathways. One unifying theme in brain disease to systemic influence and vice versa is the role of extracellular vesicles (EVs) as a means of intercellular and inter-organ communication. EVs traverse the blood–brain barrier; carry proteins, RNAs, lipids, and other cargo; and mediate glial–neuronal, endothelial–neuronal, and peripheral–CNS signaling [16,17,18,19]. For example, Lorca et al. (2024) used next-generation proteomics to examine EVs isolated from schizophrenic (SZ) brain regions [20]. In SZ brains, region-specific EV proteins were linked to disrupted GABAergic and glutamatergic signaling, synaptic loss, immune dysregulation, and cellular imbalance. The study also identified an active molecular exchange network among three brain regions—the prefrontal cortex, hippocampus, and caudate—via EVs in healthy brains, which appeared largely disrupted in SZ [20]. Thus, it can be argued that an altered EV-mediated molecular communication contributes to the pathophysiology of SZ. Additional work shows that peripheral EVs reflect and perhaps mediate CNS injury and remodeling, making them promising biomarkers and therapeutic vehicles [18,19,21,22].

In addition to local and biofluid biomarkers, neuroimaging biomarkers are increasingly used in modern medicine and research for brain disorders, and they include tools like PET scanning, MRI, etc. MRI has been a popular radiological tool that detects structural atrophy (Structural MRI) or white matter damage (Diffusion Tensor Imaging), provides biochemical information (magnetic resonance spectroscopy), and measures brain activity and assesses functional connectivity between different brain networks (Functional MRI) in a noninvasive way [23,24,25]. In this Special Issue, Martins et al. (2025) analyzed 25 studies published between 2015 and 2025 that examined resting-state fMRI (rs-fMRI) connectivity patterns in adult dyslexic patients [26]. This study is of great importance, as most studies focused on children (92%), revealing a research gap in the adult population. Functional connectivity (FC) abnormalities were primarily left-hemispheric (40%), especially in posterior regions related to phonological and visual–phonological processing. Increased right-hemispheric connectivity (20%) was interpreted as compensatory neural adaptation. Further, dyslexia affects distributed neuronal dysfunction beyond reading circuits, and, therefore, broader disruptions were also observed across executive, language, and salience networks [26]. Hence, the review emphasizes integrating rs-fMRI with cognitive assessments to clarify neural plasticity and guide intervention strategies. Another study by Ríos-Anillo et al. (2024) used MRI volumetry to examine brain structural changes in 36 individuals genetically at risk for Huntington’s disease (HD) from Colombia’s Atlántico region [27]. The AI software (Entelai/IMEXHS v4.3.4) quantified brain volumes and correlated them with CAG repeat expansions in the *Huntingtin* gene. The key findings of the study showed that the participants with ≥40 CAG repeats exhibited increased cerebrospinal fluid and amygdala volumes but marked reductions in white matter, cerebellum, brainstem, and left pallidum compared with those having <40 repeats. Thus, this study reveals early neuroanatomical alterations in at-risk individuals preceding clinical symptoms and advocates the use of AI-based neuroimaging for detecting structural biomarkers facilitating earlier diagnosis of HD and intervention up to a decade before the onset of motor defects [27].

In conclusion, the expanding molecular and biomarker toolkit from EVs, neuroimaging, and glial energetics to ionic regulation and the peripheral–brain axis has proven to be a unifying sequence in brain dysfunction across disorders. Bridging domains (psychiatry, neurology, nephrology, and vascular medicine) and leveraging shared molecular mechanisms may further accelerate biomarker development and therapeutic innovation. Our Special Issue has highlighted emergent molecular insights, illustrated cross-disease commonalities, and discussed translational opportunities. The six studies in this Special Issue highlighted herein represent the vanguard of this convergence; building on them will reveal new horizons in brain health across systemic and central disorders.
